# Artificial Intelligence in Pancreatic Cancer: From Predictive Performance to Clinical Translation

**DOI:** 10.3390/cancers18203253

**Published:** 2026-10-09

**Authors:** Ikra Khan, Azzadinne Belhaj, Alessandro Gemini, Kenza Azra Ibis, Nouman Darsif, Najoua Rouani, Aude Vanlander, Nouredin Messaoudi

**Affiliations:** Department of Hepatopancreatobiliary Surgery, Vrije Universiteit Brussel, Universitair Ziekenhuis Brussel and Europe Hospitals, 1090 Brussels, Belgium; ikrakhan@vub.be (I.K.); azzadinne.belhaj@vub.be (A.B.); alessandro.gemini@hotmail.it (A.G.); kenza.azra.ibis@vub.be (K.A.I.); nouman.darsif@vub.be (N.D.); n.rouani@cdle.be (N.R.); aude.vanlander@uzbrussel.be (A.V.)

**Keywords:** pancreatic ductal adenocarcinoma, artificial intelligence, deep learning, radiomics, computational pathology, multimodal integration, early detection, clinical translation, external validation, predictive biomarker

## Abstract

Background: Artificial intelligence (AI) is now applied across the pancreatic ductal adenocarcinoma (PDAC) pathway, yet the literature has grown faster than the evidence needed to act on it. The question is not whether an algorithm can predict an endpoint, but whether it yields information reliable, generalizable, and actionable enough to change a clinically meaningful decision. Methods: In this narrative, in a non-systematic review of PDAC-specific literature, we grade applications on an author-defined evidence-readiness framework (development, internal, external, prospective validation, clinical impact, and implementation) and on four dimensions—validation, comparator, actionability, and impact. Findings: Contemporary meta-analyses show high pooled accuracy for AI-based early detection and for distinguishing PDAC from mass-forming pancreatitis (sensitivity ~0.88–0.92, specificity ~0.90–0.93), but with extreme heterogeneity, low radiomics quality, scarce external validation, and—for electronic health-record risk models—positive predictive values often below 1% at population prevalence. Computed tomography (CT)-based detection and risk enrichment are among the more mature applications identified, yet none has been shown to improve stage distribution, resection rate, or survival. Treatment prediction is held to a strict standard, distinguishing prognostic association from a validated treatment-by-biomarker interaction. Conclusions: The rate-limiting steps are no longer algorithmic but translational—external and prospective validation, calibration, appropriate comparators, and workflow integration.

## Simple Summary

Pancreatic cancer is usually found late and remains one of the deadliest cancers, so there is intense interest in whether artificial intelligence (AI) can help find it earlier, characterize it more precisely, and guide treatment and surgery. Hundreds of studies now report that AI can predict cancer-related outcomes from scans, tissue slides, molecular data, and electronic health records, often with high reported accuracy. This review asks a stricter question: not whether an algorithm can predict an outcome, but whether it produces information reliable, generalizable, and actionable enough to change a decision that affects a patient. We grade the evidence across the whole disease pathway using an explicit “readiness” scale and four practical tests—how robustly a tool was validated, whether it was compared against the real clinical standard, whether its output would change a decision, and whether using it has been shown to improve care. We find comparatively more validation evidence for CT-based detection and risk prediction from routine records, while most other applications remain early-stage. The central message is that the main obstacles to clinical use are no longer computational; they are external and prospective validation, calibration, appropriate comparisons, and integration into everyday workflows.

## 1. Introduction

Pancreatic ductal adenocarcinoma (PDAC) remains among the most lethal common cancers, causing an estimated 467,000 deaths worldwide in 2022, with rising incidence and five-year survival still in the low teens in most high-income settings [1,2]. Stage at diagnosis dominates prognosis: most patients present with locally advanced or metastatic disease when neither surgery nor current systemic therapy is curative, whereas patients whose disease is detected at a resectable stage survive substantially longer [2,3]. Contrast-enhanced CT is the recommended first-line examination for detection, staging, and resectability, yet its interpretation is not standardized—standardized reporting templates exist but are inconsistently adopted—and small or early lesions and subtle secondary signs are frequently missed in routine practice [3,4,5,6,7]. Any technology that could move diagnosis earlier, sharpen treatment selection, or make expertise more consistent and scalable is therefore of clear interest.

Artificial intelligence (AI)—here, an umbrella term for machine learning (ML), deep learning (DL), radiomics and, increasingly, large multimodal and generative models—has been proposed for all these roles, and the literature now runs to thousands of studies. A recurring rhetorical move is to equate a high area under the receiver operating characteristic curve (AUC) on a retrospective dataset with clinical value. That equation is unsafe: diagnostic and prognostic modelling in oncology has repeatedly shown that strong internal performance can collapse on external data, that models can exploit dataset-specific shortcuts rather than biology, and that a statistically significant association with an outcome says little about whether acting on the model helps a patient [8,9,10,11]. The question governing this review is therefore restrictive: **Where can AI meaningfully change pancreatic cancer research or clinical decision-making, and how strong is the supporting evidence?**

The **primary contribution** of this review is not that it spans the continuum—recent reviews already organize pancreatic AI by the patient’s journey [12], imaging [13,14], drug discovery [15], biomarkers, and the tumour microenvironment [16]—but that it grades the field by *evidence readiness and clinical translation*. We evaluate each domain on an author-defined evidence-readiness framework, developed for this review (development → internal validation → external validation → prospective validation → clinical impact → implementation) and, orthogonally, on four dimensions—**validation, comparator, actionability, and demonstrated impact**—recognizing that a perfectly externally validated model with no actionable use has not earned clinical relevance. Full-continuum coverage and the convergence toward multimodal systems are treated as secondary, supporting features. To keep the appraisal consistent, we separate four claims that are routinely conflated—technical performance, clinical validity, clinical utility, and demonstrated patient benefit—and we keep a strict boundary between *prognostic* prediction (how a patient will fare) and *predictive* biomarkers of treatment benefit (whether a specific therapy will help) because a model associated with survival is frequently and incorrectly described as predicting response.

**Figure 1:**
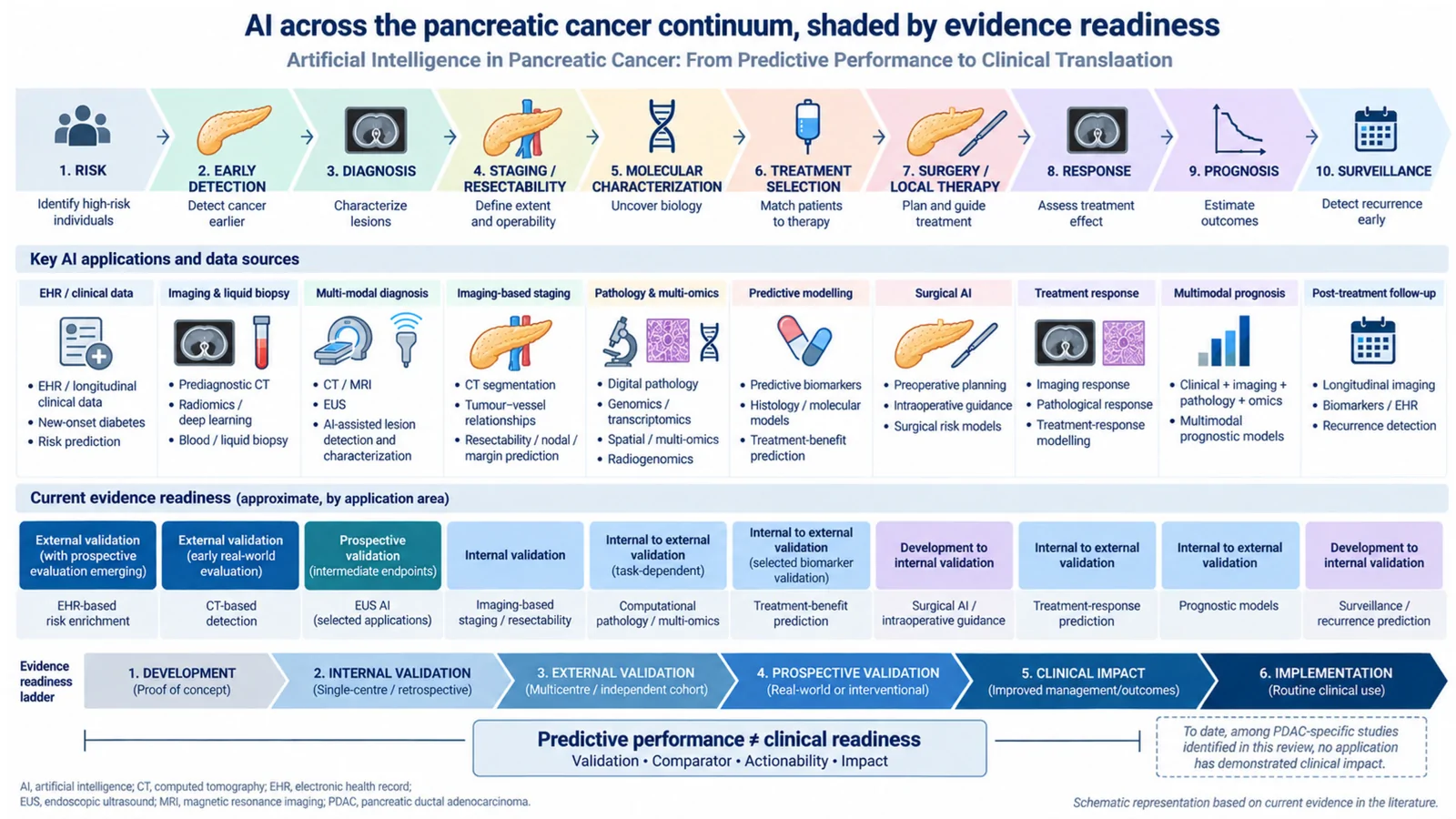
AI across the pancreatic cancer continuum, shaded by evidence readiness. AI spans the full PDAC pathway, but evidence readiness is uneven and, importantly, among the PDAC-specific studies identified in this review, no stage has reached the demonstrated clinical impact rung. Risk enrichment and CT detection are among the more mature (external and early real-world validation); treatment-response prediction, intraoperative guidance, and surveillance remain earlier. Shading uses the readiness ladder to avoid implying clinical maturity that the evidence does not support.

This review follows the clinical PDAC pathway rather than a taxonomy of algorithms, and treats PDAC as the focus, discussing other neoplasms only where they bear on differential diagnosis, precursor surveillance, or methodology. Three arguments recur. First, CT-based detection and EHR-based risk enrichment are supported by comparatively advanced validation designs among the studies identified, yet even there, to our knowledge, no PDAC-specific study has shown that deployment changes stage, resection rate, or survival. Second, aggregate accuracy—now quantifiable from several 2025–2026 meta-analyses—consistently exceeds translational maturity because external validation, calibration, appropriate comparators, and prospective evaluation are the exception. Third, multimodal integration is a prominent direction, but its value must be judged by incremental benefit over the best simple model, not by the fact that a complex model can be built. Figure 1 maps AI applications and their dominant data modalities onto this pathway, and Table 1 summarizes them together with their current evidence readiness.

## 2. Methods: Search Strategy and Scope

This is a narrative review and does not follow a systematic review (e.g., PRISMA) protocol; it makes no claim of exhaustive or reproducible study capture, and the selection of illustrative studies reflects expert judgement. To make the basis of that selection transparent, we searched PubMed/MEDLINE, Web of Science, Scopus, and Google Scholar for literature on AI in pancreatic cancer, prioritizing work published between January 2022 and 2026 and supplementing it with seminal older studies where they defined a method, benchmark, or clinical concept. Search terms combined “pancreatic cancer”, “pancreatic ductal adenocarcinoma” and “PDAC” with “artificial intelligence”, “machine learning”, “deep learning”, “radiomics”, “radiogenomics”, “computational/digital pathology”, “multi-omics”, “multimodal”, “foundation model”, “large language model”, “generative AI”, and domain-specific clinical terms (early detection, EUS, resectability, postoperative pancreatic fistula, treatment response, prognosis). Citation chaining from recent meta-analyses, consensus statements, and landmark primary studies was used to identify work that keyword searches missed. We drew on two complementary bodies of evidence rather than a rigid hierarchy: systematic reviews and meta-analyses were used to establish aggregate, quantitative performance across studies, while recent multicentre, externally validated or prospective primary studies—together with landmark studies that define a subfield—were used to establish the frontier and the readiness level of each application. Narrative reviews were used to map the field and locate primary literature, not as evidence of performance. References supporting key numerical, comparative, predictive, or maturity claims were checked against their primary sources, and citations that could not be verified were removed rather than retained. Because the search was deliberately non-exhaustive, statements about the absence of evidence should be read as applying to the PDAC-specific literature identified for this review rather than as formal proof of absence.

To make the basis of selection transparent while remaining faithful to a narrative design, we applied the following criteria. Eligible sources were English-language, peer-reviewed studies and evidence syntheses addressing AI or machine learning applied to PDAC across the disease pathway; we gave less weight to conference abstracts without full texts and to studies of non-pancreatic cancers except where methodologically illustrative. The database-specific strategies are provided in Supplementary Table S1; these strategies were reconstructed and run for transparency during the revision, with the most recent search performed in October 2026, and are not presented as a prospectively preserved search string or as a systematic-review protocol. Study identification and selection were carried out by members of the author team as part of an expert-curated process rather than by independent dual screening against a registered protocol, and the number of records screened was not prospectively recorded; where several reports described the same cohort, the most complete or most recent was used. We treated a study as landmark when it defined a method, benchmark or clinical concept, achieved multicentre, external or prospective validation, or was the largest or most rigorous example in its domain. To limit the selection bias inherent in an expert-curated narrative review, we drew on systematic reviews and meta-analyses for quantitative statements wherever possible, treated readiness designations as author interpretation, checked key reported performance figures against their primary sources, and framed absence-of-evidence statements as applying to the identified literature rather than as proof of absence. A conceptual, non-numeric schematic of the (non-systematic) selection process is provided in Supplementary Figure S1.

## 3. From Algorithms to Clinical Questions: An Evidence-Readiness Framework

A short orientation to terminology is warranted because the same words carry different weight in computational and clinical settings. Machine learning models learn statistical mappings from data; classical approaches (logistic regression, support vector machines, random forests, gradient boosting) typically use predefined features and remain competitive, interpretable baselines. Deep learning uses multilayer neural networks that learn features directly from raw inputs such as CT voxels or whole-slide images; convolutional and, increasingly, transformer architectures dominate imaging applications [8,17]. Radiomics extracts high-throughput quantitative image features that are analyzed with ML, and a consequential distinction—developed in Section 6—separates reproducibility-testable handcrafted radiomics from more expressive but opaque deep learning features. Foundation models are large networks pretrained by self-supervision and adapted to specific tasks with limited labels.

What matters clinically is less the architecture than the structure of the question and the standard of proof applied to the answer. A recurring set of questions runs across the pathway—does a lesion exist (detection), where and how large (segmentation), what is it (differential diagnosis), how advanced and resectable (staging), what is its biology (molecular characterization), how will the patient fare (prognosis), and, most demanding, which treatment will help (prediction of benefit)—and each has a natural clinical comparator: a radiologist or pathologist, TNM stage, CA 19-9, an established clinical risk score, or the multidisciplinary tumour-board decision. We appraise every major application on an author-defined **evidence-readiness framework**—development, internal validation, external validation (temporal, geographic, single-centre, multicentre, or cross-country), prospective validation, demonstrated clinical impact, and routine implementation—and, orthogonally, on four dimensions: **validation** (how robustly the output has been shown to hold in the intended-use population), **comparator** (whether it was benchmarked against the true clinical standard rather than a weak or absent baseline), **actionability** (whether the output would change a decision), and **impact** (whether using it has been shown to improve care). An overall maturity designation is assigned only after those four dimensions are assessed because they are not interchangeable—a well-externally validated survival model with no actionable consequence is not clinically useful, and a highly actionable output that has never been externally validated is not yet trustworthy. Table 2 summarizes the landmark and recent high-impact studies that anchor this appraisal, and Table 3 applies the framework across the major application areas. This framework is a pragmatic synthesis developed by the authors for this review rather than a validated staging instrument; its rungs are defined in Figure 1 and applied consistently in Table 3.

Metric discipline is part of the same framework. Discrimination (AUC, sensitivity, specificity) says whether classes can be separated but nothing about whether probability outputs are trustworthy or whether acting on them helps. Calibration, which is the agreement between predicted and observed risk, governs whether scores can be used at a decision threshold, and a well-discriminating but poorly calibrated model can be harmful if taken at face value. Decision-curve analysis asks whether using the model across plausible thresholds beats default strategies. For screening, positive and negative predictive values at the true intended-use prevalence, rather than the inflated prevalence of a case–control set, decide feasibility. The pancreatic AI literature reports AUC almost universally and calibration or decision-curve analyses rarely. Throughout this review, we weight a calibrated, decision analytically evaluated, externally validated model far above a higher-AUC model lacking those properties, and we treat AI-specific reporting standards (e.g., STARD-AI, transparent challenge reporting) as the baseline expectation rather than an optional extra [37,38]. The near-absence of calibration and decision-curve reporting is itself a major evidence gap rather than a minor omission, and we flag it as such wherever it bears on a claimed clinical use. The effect of pre-test prevalence on positive and negative predictive values is illustrated in Table 4.

## 4. AI for Biological Discovery and Translational Research

It is useful to organize AI in PDAC discovery by function, including representation learning and subtype discovery, cell-state and spatial inference, target identification, drug-response modelling, and drug discovery/repurposing with functional validation, and, in doing so, to be explicit in that much of what is labelled “AI” in this space is established computational biology (unsupervised clustering of transcriptomes, differential expression, and single-cell analysis) rather than a novel algorithmic advance. The distinction matters because reviewers rightly object when routine bioinformatics is rebranded, and because the genuine AI contribution here is narrower and more specific than the enthusiasm suggests.

The molecular architecture of PDAC, such as near-ubiquitous KRAS, TP53, CDKN2A, and SMAD4 events in a dense immunosuppressive stroma, and reproducible transcriptomic subtypes, most durably the basal-like (squamous) versus classical axis with stromal programmes, was established largely by conventional genomics and proteogenomics [3,39,40]. Against that backdrop, AI’s contribution to subtype discovery is chiefly to improve robustness and cross-cohort reproducibility of classification: multi-omic ensemble ML can recover and stabilize subtypes and derive prognostic signatures validated across external cohorts [41]. What such studies rarely establish is incremental actionability—whether a newly derived AI subtype changes management beyond the existing basal-like/classical scheme. Therefore, most rest at internal or external validation on the readiness ladder but score low on actionability and impact. AI is more distinctively enabling at cell-state and spatial inference, where computational methods are required simply to make single-cell and spatial data tractable; single-cell work has charted the trajectory of tumour-infiltrating lymphocyte states that helps explain why an apparently T-cell-containing PDAC remains immunologically inert [42]. The most translationally oriented discovery work couples these analyses to target identification and drug repurposing: an integrative pipeline combined single-cell multi-omics, ML prognostic modelling and structure-based virtual screening to nominate an IGF2BP2-high malignant state as a driver of microenvironmental immunosuppression and to repurpose montelukast as a functional inhibitor that restored CD8^+^ T-cell cytotoxicity in vitro [43]. The study is a clean template for computational discovery, but its evidence is explicitly preclinical and single-cell-line, and the working drug concentrations exceeded clinically achievable plasma levels—an honest marker of the distance between an AI-nominated target and a usable therapy. Across the discovery domain, then, AI is a mature accelerator of hypothesis generation whose outputs, such as targets, signatures, and mechanistic hypotheses, remain early on the readiness ladder and reach patients only through the same experimental and trial bottlenecks as any other preclinical finding.

The *drug discovery and repurposing* axis is where the distinctively new AI methods concentrate, and where the gap between computational promise and clinical proof is widest. Structure-based deep learning for protein-structure prediction, large-scale virtual screening, and generative molecular design are being applied to PDAC’s historically “undruggable” targets—most prominently the near-ubiquitous *KRAS* oncoprotein, where AI-assisted medicinal chemistry is now interleaved with the emerging G12C, G12D, and pan-*KRAS* inhibitor programmes [15]. These pipelines can nominate candidate chemotypes and repurposable agents far faster than conventional screening, but the same reviews are candid that PDAC’s dense stroma, metabolic plasticity, and rapid, heterogeneous resistance blunt single-agent activity; therefore, an AI-nominated molecule still faces the field’s ordinary attrition. Patient-derived organoids and other functional models are increasingly used as the experimental read-out that grounds these predictions, providing a chemosensitivity phenotype against which a computational signature can be tested before it is ever taken toward trial. The honest summary of the discovery domain is therefore asymmetric: AI meaningfully compresses the hypothesis-generation and candidate-prioritization steps, but it does not shorten the experimental validation, safety, and trial pathway that separates a target from a therapy, and no PDAC target or repurposed agent surfaced primarily by these methods has yet changed clinical practice.

## 5. Risk Prediction and Early Detection

Early detection is where AI has a strong theoretical case and, among the studies identified in this review, comparatively advanced evidence. However, it is also where conceptual precision matters most. Four tasks are routinely conflated: *risk enrichment* (raising a subgroup’s pretest probability enough to justify testing), *screening* (applying a test to that group), *early detection* (finding subclinical disease), and *diagnosis* (confirming it). Because PDAC prevalence is low even in enriched groups, screening demands exceptional specificity, and performance must be reported at the intended-use prevalence rather than the inflated prevalence of a case–control set.

The aggregate evidence is now quantifiable and instructive. A 2026 meta-analysis of radiomics for early PDAC detection pooled 15 studies and 14,688 patients and reported a sensitivity of 0.88 (95% CI 0.84–0.91) and a specificity of 0.93 (0.87–0.96)—but with I^2^ of 88–95% and an explicit call for prospective evaluation [18]. A 2026 meta-analysis restricted to EHR-based AI risk models found a pooled AUC of 0.785, with neural networks highest (0.826) but positive predictive values consistently below 1% at population prevalence and extreme between-study heterogeneity—precisely the profile expected when screening a low-prevalence disease, and a direct warning against general-population deployment [20]. A 2026 meta-analysis of liquid-biopsy-plus-AI for early cancer detection (pancreatic *n* = 16) found high specificity (94–99%) but stage I sensitivity ranging from 44–91% [21]. The consistent message across syntheses is that headline accuracy is high but heterogeneous, methodological quality is moderate, external validation is scarce, and no modality yet meets population-screening requirements; the credible path is integration within defined high-risk cohorts [18,20,21,44].

Landmark studies show why. A prominent risk-enrichment tool operates on routine clinical data: a transformer model trained on diagnostic-code sequences from ~6 million people in Denmark and ~3 million in the US Veterans Affairs system predicted incident PDAC within 36 months at an AUC of 0.88 (0.83 when events in the final 3 months were excluded), and—critically for this review’s thesis—cross-country application degraded performance to an AUC of 0.71 and required retraining, a concrete demonstration of dataset shift across health systems [24]. A mechanistically grounded enrichment strategy targets new-onset diabetes: the transparent END-PAC score (weight change, glycaemia, and age) identified 3-year PDAC risk at an AUC of 0.87 in its discovery cohort and now anchors the prospective Early Detection Initiative trial—one of the few examples in the field of progression from a simple model toward prospective evaluation [25,45]. On imaging, radiomics-based ML detected a signature of carcinogenesis on prediagnostic CTs that appeared normal to experts, at an AUC of 0.98 versus 0.66 for two radiologists at a median of an ~400-day lead time [46]. An automated 3D-CNN reached a diagnostic AUROC of 0.97 and detected visually occult preinvasive PDAC on prediagnostic CTs at an AUROC of 0.91 (sensitivity 0.75, specificity 0.90) at a 475-day median lead time, with external public-dataset validation and bootstrapped testing at 1–5% simulated prevalence [26]. Its successor, REDMOD, was explicitly built and tested in a simulated ~1:6 low-prevalence setting, reaching an AUC of 0.82 and 73% sensitivity (roughly twice that of the radiologists’) with longitudinal stability and external specificity validation [27]. These three imaging studies are methodologically rigorous, but all are retrospective and case–controlled (case-enriched), and none has been evaluated prospectively at true screening prevalence; automated, externally validated pancreas segmentation—now at Dice ~0.94–0.96—removes a practical barrier to scaling them but does not change their readiness level [47]. Fluid-based approaches provide an orthogonal axis: multi-analyte blood tests localize resectable cancers, including PDAC [48]; multimarker panels guide cyst management [49]; CA 19-9 trajectories show pre-diagnostic lead-time signals [50]; and methylation-based multi-cancer early-detection assays report high specificity with still-limited early-stage sensitivity [51]. Placed on the readiness ladder, EHR risk enrichment and CT-based detection are among the more mature applications identified (external/real-world validation, one prospective trial in progress), while prediagnostic radiomics and circulating markers remain externally validated at best, and unproven at true prevalence.

## 6. AI in Pancreatic Imaging

Imaging has produced some of the field’s most rigorously validated PDAC evidence, but two flagship studies answer different questions and should not be conflated. **PANDA** addressed detection on *non-contrast* CT: trained on 3208 patients and validated across 6239 patients from 10 centres (lesion-detection AUC 0.986–0.996), it outperformed the mean radiologist by 34.1% in sensitivity and 6.3% in specificity for PDAC identification and reached 99.9% specificity in a **retrospective** real-world evaluation of 20,530 consecutive patients—performance that makes opportunistic screening on routine scans conceivable [22]. **PANORAMA** addressed benchmarking rigour on *contrast-enhanced* CT: an open, multicentre benchmark with the largest reader study to date (68 radiologists, 12 countries) in which the AI system reached an AUC of 0.92 versus 0.88 for the radiologist pool, meeting prespecified non-inferiority and superiority criteria while producing fewer false-positive findings [23]. Together, these are among the more mature validation evidence for PDAC imaging that AI identified in this review—multicentre, reader-benchmarked, and, for PANDA, real-world. However, PANDA’s real-world study remains retrospective, and PANORAMA is a controlled reader study; neither demonstrates that deployment improves stage distribution, diagnostic delay, resection rate, or survival—an impact endpoint we did not find demonstrated in the PDAC imaging literature identified for this review. Both author groups state that prospective, workflow-integrated validation is the necessary next step, and PANORAMA’s central methodological argument—that proprietary data and non-standardized evaluation have held the field back—is itself an important correction [23]. Aggregate diagnostic performance for AI on pancreatic imaging is high (a 2023 meta-analysis of seven EUS-based AI studies in 1110 patients reported pooled sensitivity of 0.93, specificity of 0.90 and an AUC of 0.95), but rests on few studies and heterogeneous designs [52], and a 2025 meta-analysis of radiomics distinguishing PDAC from a mass-forming pancreatitis (24 studies, 14,406 cases) reported pooled sensitivity of 0.92 and specificity of 0.90 while documenting a mean radiomics-quality score of only ~9/36 and pervasive deficiencies in external validation, standardization, and reproducibility [19]—again, accuracy outrunning maturity. The single-centre nationwide and cross-racial detection studies that populate this literature are technically strong but heterogeneous in design and comparator [53,54,55], and the field’s maturation is visible in its methodological borrowing: the paired, non-inferiority, confirmatory design of PANORAMA mirrors the PI-CAI prostate-MRI study, and the move toward open FAIR benchmarks reflects a recognition that dataset quality gaps limit reproducibility [56,57,58].

Two distinctions recur. First, **handcrafted radiomics versus deep learning features:** Handcrafted features are explicit and, in principle, reproducible, but their reproducibility is contingent on standardized extraction and is measurably sensitive to slice thickness, reconstruction kernel, and voxel size, and confounder-driven “signatures” have been formally demonstrated [11,58]. However, deep learning features are more expressive but opaque and data-hungry and can learn dataset-specific shortcuts unless trained on large, diverse data, which is one reason why the most robust CT systems rely on thousands of multicentre studies [22,23]. Second, **diagnostic versus radiogenomic** readouts: Predicting molecular features (subtype, mutations, and proliferation) from images is attractive, but in PDAC, it remains at the internal validation stage on small cohorts and should be treated as hypothesis-generating. MRI and PET add complementary information and are the subject of growing DL work, but we identified no MRI/PET tool for PDAC that has reached the evidentiary level of the leading CT systems. For staging and resectability—decisions that directly determine whether a patient is offered surgery—AI segmentation can quantify tumour–vessel contact and classify resectability with roughly 75–85% concordance with expert radiologists, and radiomic models predict nodal involvement and margin statuses preoperatively; these outputs remain at the internal validation stage and are not yet embedded in multidisciplinary decision-making [59]. This is the imaging decision with the most direct consequence for the patient—it determines whether surgery is offered, whether neoadjuvant therapy precedes it, and whether an operation would be futile—so the comparator that matters is not a single radiologist but the multidisciplinary assessment that integrates arterial and venous contact, tumour–vessel interface length and morphology, and response to induction therapy. Among the studies identified here, no AI tool has yet been shown to change that assessment or its downstream operative decisions—an impact endpoint that the resectability literature still lacks.

MRI and PET extend the imaging repertoire but trail CT in evidentiary maturity. Diffusion-weighted and contrast-enhanced MRI are advantageous for small or isodense lesions, for characterizing cystic precursors, and for liver metastasis detection, and deep learning work on these sequences is growing; PET and PET/MRI add functional and metabolic information relevant to distinguishing viable tumours from post-treatment changes and to detecting occult metastatic disease. In PDAC, however, we identified no MRI or PET AI tool that has reached the multicentre, reader-benchmarked evidentiary level of the leading CT systems, and much of the work remains single-centre and retrospective. The near-term contribution of MRI- and PET-based AI is therefore best framed as complementary problem-solving—resolving the equivocal CT finding and characterizing the indeterminate cyst or liver lesion—rather than as a competing front-line detector, and it should be held to the same external validation and comparator standards applied to CT.

Endoscopic ultrasound (EUS) is the imaging modality closest to AI-assisted deployment because it is operator-dependent and therefore stands to gain most from consistency, and, unusually, it has generated prospective randomized evidence. Convolutional neural network systems differentiate PDAC from chronic and autoimmune pancreatitis and from neuroendocrine tumours [60], a real-time biliopancreatic EUS device improved lesion recognition in a single-centre randomized controlled trial [61], and a multimodal AI system was tested in a randomized crossover trial for solid pancreatic lesions [62]. EUS thus illustrates both the promise and the ceiling of current work: genuine prospective testing exists but is confined to intermediate endpoints (detection, characterization) at single centres rather than to demonstrated effects on management or outcomes [63].

A recurring differential diagnostic challenge is distinguishing solid pseudopapillary neoplasm (SPN)—a low-grade, usually curable tumour of young women—from PDAC, neuroendocrine tumours, and cystic lesions because misclassification drives very different management. Current reviews summarize SPN diagnosis as resting on characteristic CT and MRI appearances with an endoscopic ultrasound-guided biopsy for confirmation [64]. Small single-centre studies suggest that MRI- and CT-based radiomics can separate SPN from these mimics (with a reported area under the curve up to ~0.96) and can estimate SPN invasiveness preoperatively [65,66]. These findings are preliminary: SPN is rare, so cohorts are small (often fewer than 25 SPN cases); designs are retrospective; single-institution, external validation is absent; and class imbalance inflates apparent performance. As with PDAC detection, the appropriate comparator is expert radiological assessment with EUS-guided tissue sampling, and no SPN-focused AI tool has yet been prospectively validated or shown to change management. The role of AI here is best framed as hypothesis-generating triage support rather than a diagnostic arbiter.

## 7. Computational and Digital Pathology

Histopathology is both the diagnostic ground truth for PDAC and a rich substrate for computation, and digital pathology has moved from tumour detection toward biologically and clinically meaningful readouts. A well-developed example of clinical relevance is spatial immune phenotyping: an AI whole-slide analyzer applied to 304 resected PDACs classified tumours as immune-inflamed, immune-excluded, or immune-desert directly from haematoxylin–eosin slides, and immune phenotype was an independent predictor of overall and recurrence-free survival after adjustment for stage, with stage II immune-inflamed disease outperforming stage I non-inflamed disease [28]. Because it needs only routine H&E, this removes the labour and inter-observer variability that have kept manual tumour-infiltrating-lymphocyte scoring out of practice, and it is a credible complement to TNM staging—but it is single-centre, upfront-resection, and treated with now-dated adjuvant regimens, and prospective, biopsy-applicable validation is absent. Therefore, on the readiness ladder, it is externally unvalidated with moderate actionability. Related computational work resolves the microenvironment into cellular neighbourhoods that coordinate the outcome, deepening interpretation of what the phenotype classifiers detect [42,67]. Toward treatment-relevant outputs, AI segmentation of residual tumour in post-neoadjuvant resection specimens has been standardized and internationally validated, addressing genuine pathologist disagreement [68]. The general trajectory—molecular-phenotype prediction from morphology, and pathology foundation models pretrained by self-supervision on large slide collections to improve cross-laboratory generalization—is promising, but most PDAC pathology-AI remains retrospective, single-institution, and dependent on scanner- and stain-specific pipelines. Automated grading and tumour detection are technically solved but are of modest clinical consequence relative to immune phenotyping or response readouts. As in imaging, the binding constraints are external validation across laboratories and prospective demonstration that acting on a computational readout changes the care path.

Two further pathology applications deserve regulated appraisal because they are frequently over-sold. First, *automated grading and tumour detection*: convolutional and transformer models now segment tumour epithelium and grade differentiation from whole-slide images with high concordance to pathologists, but this addresses a task that expert pathologists already perform reliably. Therefore, its clinical value is efficiency and standardization rather than a new capability, and it should not be presented as equivalent in consequence to immune-phenotyping or response read-outs. Second, *molecular-phenotype prediction from morphology*—inferring transcriptomic subtype, specific mutations, or a treatment-relevant programme directly from H&E—is attractive because it would extract molecular information without sequencing, but in PDAC, it remains at internal validation on modest single-institution cohorts and is best treated as hypothesis-generating until externally validated against the molecular ground truth it claims to predict. Pathology foundation models, pretrained by self-supervision on very large slide collections and then adapted with limited PDAC-specific labels, are a promising route to the cross-laboratory generalization that the field lacks, and the treatment-relevant, internationally validated residual-tumour segmentation tool [68] shows that consortium-scale annotation and multi-scanner training can produce a genuinely deployable read-out. However, for most pathology AI, the decisive, still-unmet requirement is prospective evidence that acting on the computational output changes a management decision rather than merely reproducing one a pathologist would have made.

## 8. Multimodal and Multi-Omics AI

**Figure 2:**
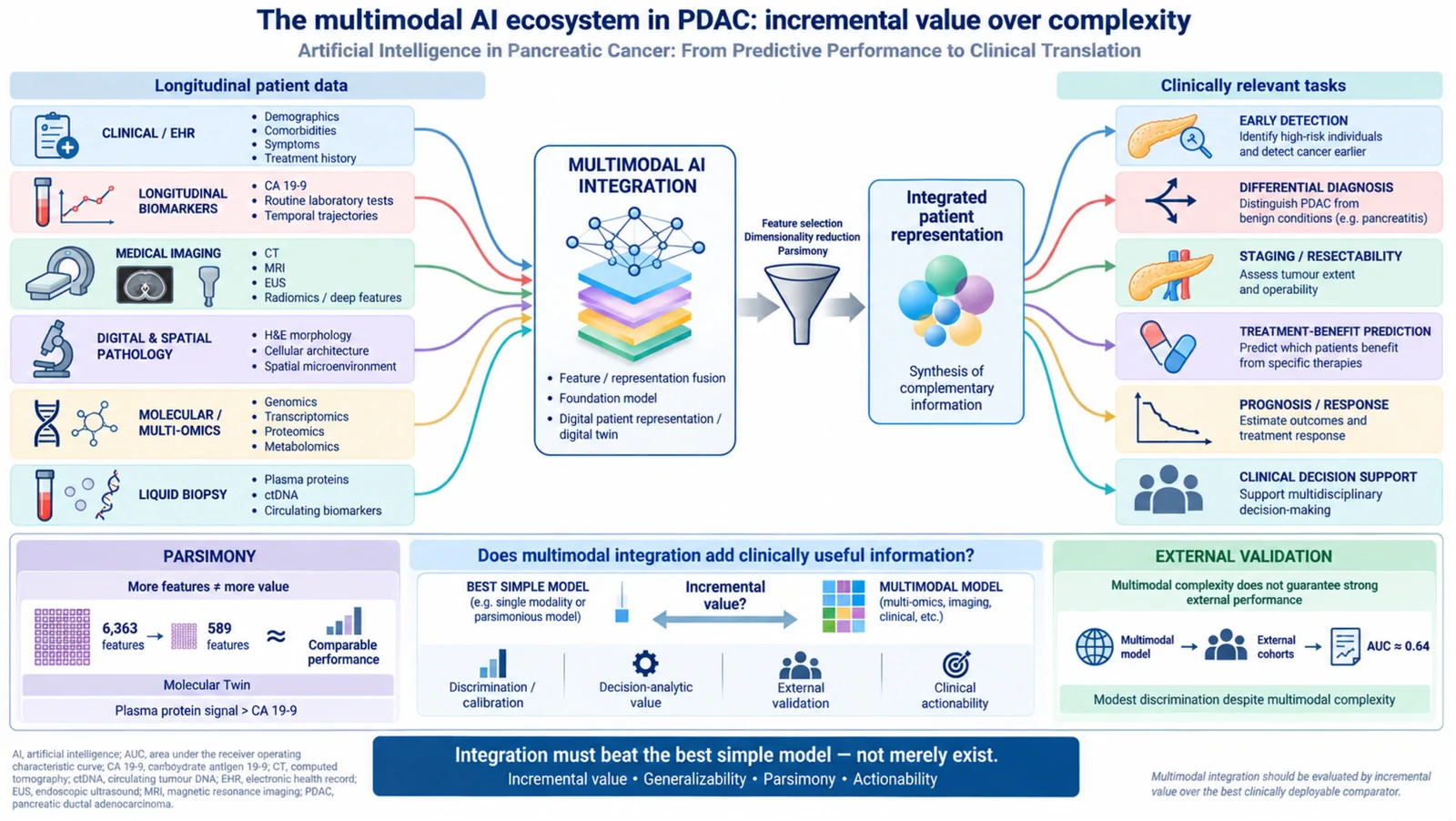
The multimodal AI ecosystem in PDAC, judged by incremental value. Pancreatic AI is converging from single-modality models toward multimodal patient representations. The decisive question is incremental value over the best simple comparator; evidence to date shows parsimonious multimodal panels can match far larger ones, and that external validation of multimodal models can still yield modest discrimination—arguing for the smallest deployable analyte set rather than the largest.

A prominent directional trend in pancreatic AI is the move from single-modality prediction toward systems integrating clinical, longitudinal, imaging, pathological, and molecular data (Figure 2)—but the honest test of multimodality is incremental value over the best simple model, not the fact that a complex model can be built. An instructive example is the Molecular Twin platform, which assembled 6363 clinical and multi-omic features (targeted DNA, whole-transcriptome RNA, tissue and plasma proteomics, lipidomics, and computational pathology) from resected PDAC to predict disease survival. This was externally validated across four cohorts [29]. Three findings should shape expectations: a full multimodal model was most accurate, confirming complementarity; a *parsimonious* 589-feature model matched the full 6363-feature model, and plasma proteins alone were the single most predictive analyte, outperforming CA 19-9. The practical implication is that the goal of multimodal work should be the smallest deployable analyte set that captures the achievable signal, not the largest [29]. A recent multimodal DL prognostication study is a useful corrective on expectations: integrating clinical variables with contrast-enhanced CT, it beat TNM and unimodal models yet achieved only modest discrimination (AUC 0.637 internally; 0.571–0.675 in Dutch and Spanish external cohorts)—an honest demonstration that multimodal integration with genuine external validation can still yield limited, if real, gains [30]. At the frontier, a Bayesian foundation-model “digital twin” outperformed conventional Moffitt stratification for predicting a modified-FOLFIRINOX response specifically within the basal-like subtype (AUC 72.3% vs. 44.8%) while performing comparably in the classical subtype—a real but subgroup- and regimen-confined gain in small cohorts [33]. Multimodal work is among the more credible medium-term directions, but must be held to the incremental-value standard.

The practical obstacles to multimodal AI in PDAC are as instructive as its promise, and they explain why incremental value is often marginal. Fusing modalities requires that images, slides, molecular assays, and longitudinal clinical data be spatially or temporally aligned and jointly represented, yet PDAC cohorts rarely have all modalities for all patients: the *missing-modality* problem forces either exclusion of incomplete cases (shrinking already-small samples and inducing selection bias) or imputation (which can fabricate signals). High-dimensional molecular features vastly outnumber patients, so naïve fusion invites overfitting and feature selection leakage unless dimensionality is aggressively and honestly controlled—precisely the discipline the Molecular Twin’s parsimonious model exemplifies [29]. Just as important is to consider that the correct comparator for a multimodal model is not just “no model” but the best available *simple* model. This is because much of what imaging or pathology contributes is already captured by stage, grade, and CA 19-9—a fair evaluation reports incremental discrimination and, ideally, decision-curve analysis over that baseline. When such comparisons are made, the gains are real but modest—an externally validated multimodal prognostic model still only reached an AUC of ~0.64 [30]—which argues for the smallest deployable, routinely available analyte set rather than the most elaborate one. Multimodality is among the field’s more credible trajectories precisely because PDAC biology is distributed across scales that no single modality captures. However, its clinical case will be made by disciplined, externally validated, incremental-value studies, not by demonstrations that a complex model can be assembled. For most PDAC multimodal models, calibration and decision-curve or net-benefit data are not reported; where a study did not report an incremental-value metric (a change in AUC or C-index, calibration change, or net benefit over the best simple comparator), we treat it as not reported rather than inferring a value.

## 9. AI for Precision Treatment and Response Prediction

Treatment selection is where the prognostic-versus-predictive distinction is most consequential and most often violated, and this section applies that distinction strictly (Box 1). First-line therapy for advanced PDAC is a choice between FOLFIRINOX-type regimens and gemcitabine/nab-paclitaxel, currently made on performance status rather than tumour biology; therefore, a genuinely predictive biomarker would be of immediate value [2,4]. A model associated with better survival among patients who happened to receive a therapy is *prognostic*; only a demonstrated *treatment-by-biomarker interaction* supports a *predictive* claim; and a retrospective biomarker analysis of a randomized-trial cohort, while stronger than an observational association, is not equivalent to a prospective biomarker-guided trial. These three tiers should be kept distinct when reading the literature.

Box 1 **Prognostic versus predictive—the distinction that governs treatment-selection AI.**

Prognostic marker: associated with the outcome (e.g., survival) regardless of the therapy; it informs what to expect, not which treatment to choose.
Predictive biomarker: identifies patients who benefit more (or less) from a specific therapy; it requires a treatment-by-biomarker interaction, ideally demonstrated within a randomized comparison of treatments.
Evidence hierarchy for a predictive claim: (1) association with an outcome among treated patients (weakest); (2) a significant treatment-by-biomarker interaction in retrospective analysis of a randomized-trial cohort (stronger, but still retrospective); (3) a prospective, biomarker-guided randomized trial (definitive).
A model that predicts survival among patients who happened to receive a drug is prognostic, not predictive; describing it as predicting treatment benefit is a category error that recurs throughout the pancreatic cancer AI literature.

Transcriptomic tools have advanced along this hierarchy. GemPred was developed to predict adjuvant gemcitabine (GEM) sensitivity [69]. Pancreas-View extended this to drug-specific signatures for each modified FOLFIRINOX (mFFX) component and was clinically validated in tissue from the randomized PRODIGE-24/CCTG PA6 trial (*n* = 343), where patients predicted sensitive to their administered regimen had markedly longer disease-free survival (median 50.0 months in the mFFX-sensitive group treated with mFFX, stratified HR 0.31; 33.7 months in the GEM-sensitive group treated with GEM, HR 0.40; versus ~10.6–10.8 months in mismatched or pan-resistant patients) [31]. This is trial-derived retrospective validation—compelling, but not a prospective biomarker-guided trial—and Pancreas-View has not been deployed prospectively as a decision aid. A methodological exemplar for a *predictive* claim is a histology-based DL biomarker (PANCprAId) that estimated relative benefits from adjuvant GEM versus mFFX: trained on 231 patients and externally validated in PRODIGE-24/PA6 (*n* = 313), its regimen-specific scores stratified outcomes within both the GEM-treated (HR 1.69) and mFFX-treated (HR 2.02) arms and, when combined, showed **significant treatment interactions for disease-free survival (interaction *p* = 0.003) and cancer-specific survival (*p* = 0.001)** [32]. A formal interaction test on randomized-trial tissue is precisely the design that distinguishes a predictive biomarker from a prognostic one, setting the standard that the rest of the field should meet—while retrospective validation still awaits prospective confirmation. Imaging is entering the same space: a CT-radiomics model of tumour fibrosis predicted response to gemcitabine/nab-paclitaxel (longer progression-free and overall survival) but not to FOLFIRINOX or SOXIRI, a treatment-specific (and therefore predictive-flavoured) signal, though with a modest external AUC (0.72) and requiring prospective testing [70]. Real-world transcriptomic models trained on large clinico-genomic databases (e.g., 2202 advanced-PDAC patients with propensity-matched, time-to-next-treatment outcomes) generate regimen-specific recommendations but inherit confounding-by-indication, which propensity matching mitigates rather than eliminates [71]. Clinico-genomic work has shown that *KRAS* mutant-allele dosage is prognostic of survival, refining stratification [72]. For the small actionable-alteration subgroups (mismatch-repair deficiency, *BRCA/PALB2*, *NTRK/NRG1* fusions, and emerging *KRAS* G12C/G12D and pan-*KRAS* inhibitors), AI’s near-term value is less about predicting response than about ensuring these rare, high-impact alterations are detected and surfaced for confirmatory testing [15,72]. The appropriate reading is cautiously optimistic: the best tools are now designed and, in the best-validated case, have been shown to be predictive rather than merely prognostic. However, to our knowledge, none has yet been evaluated prospectively as a decision aid, and describing any as ready to guide therapy selection would overstate the evidence.

## 10. AI in Pancreatic Surgery and Perioperative Care

Surgery is the only potentially curative treatment for PDAC, and its AI evidence is best evaluated in three phases of differing maturity. Preoperatively, segmentation models quantify tumour–vessel contact and classify resectability, and radiomic models estimate nodal and margin risks, informing the neoadjuvant-versus-upfront decision. These remain at the internal validation stage and are not integrated into tumour-board workflows [59]. DL prediction of overall survival after resection has been developed and multicentre-tested, offering stratification beyond stage [73]. Postoperatively, complication prediction is the most concrete application: an ensemble model combining clinical, body composition, and CT-derived DL features predicted postoperative pancreatic fistula (POPF) preoperatively with an AUC of 0.75 (0.68 for clinically relevant POPF) in 1333 pancreatoduodenectomies [34]. A registry model predicted early death (“futile” surgery, death within 12 months) after pancreatoduodenectomy in 66,331 patients with an AUC of 0.689 [35]. Both are modest in discrimination. As such, a critical, still-underdeveloped point is that many surgical-AI papers are benchmarked against weak baselines when they should be compared against established instruments such as the (alternative) Fistula Risk Score for POPF or validated prognostic staging for survival. Without such a comparator, incremental value is unproven. Intraoperatively, “surgomics” frameworks aim to fuse intraoperative video and sensor data with preoperative information [74]. Furthermore, computer-vision methods for phase recognition, landmark detection, and augmented reality guidance are adapted from higher-volume procedures, but pancreatic-specific intraoperative evidence is essentially proof-of-concept because pancreatoduodenectomy is lower-volume and more anatomically variable than cholecystectomy and colorectal procedures, where surgical video AI has advanced. Finally, large language models have been evaluated as tumour-board decision support: assessed against multidisciplinary decisions in 42 PDAC cases, an LLM’s performance depended heavily on prompt design and diverged from expert reasoning at the hardest junctures, such as vascular encasement and resectability, equivocal metastatic findings, and the recognition of neoadjuvant therapy as a curative pathway, indicating its role in structuring information rather than substituting surgical judgement [36]. A realistic near-term contribution across the phase is decision support, and evaluation should shift from AUC toward whether these tools change recommendations or perioperative management.

## 11. Prognostication, Recurrence and Surveillance

Prognostic modelling is the most crowded and least differentiated area of pancreatic AI, because survival is easily associated with almost any rich data source. Recurrent failure is not a matter of stratification but of the absence of two things: a formal test of *incremental value* beyond the established clinicopathological model (such as stage, margin, grade, nodal status, and CA 19-9), and respect for the prognostic-versus-predictive boundary. The most rigorous recent contributions are those with an interpretable readout and external validation, such as the H&E-based immune-phenotype classifier that independently predicts survival in resected disease [28], the multimodal Molecular Twin with plasma protein outperforming CA 19-9 [29], and externally validated multimodal DL prognostication that beats TNM, albeit with a modest AUC [30,73]. Radiomic and pathology signatures for recurrence and patterns are numerous but predominantly single-centred and, as such, are rarely benchmarked head-to-head against standard models with decision-curve analysis. For longitudinal surveillance—arguably where AI could most plausibly help by detecting recurrence earlier on serial imaging or integrating CA 19-9 trajectories—prospectively evaluated tools are essentially absent, causing a clear gap between demonstrated prediagnostic feasibility [27,46] and any validated surveillance application.

Two distinctions refine this appraisal. First, predicting *whether* a patient will recur is less clinically useful than predicting *where* and *when*: isolated local recurrence, oligometastatic liver disease, and diffuse peritoneal spread carry different management implications. Yet, most recurrence models predict a single time-to-event endpoint and are rarely benchmarked head-to-head with the established clinicopathological model using formal tests of incremental value or decision-curve analysis. A model that reproduces the stage, margin, grade, nodal status, and CA 19-9 only increases costs and opacity without contributing to decision-making. Second, surveillance is the setting in which the prediagnostic-detection work reviewed in Section 5 is most directly transferable—the same longitudinal imaging and biomarker trajectory methods that flag subclinical primary disease could, in principle, flag early recurrence in a resected cohort at a genuinely elevated pretest probability. However, this transfer has not been made: the prediagnostic models were trained and tested for an initial diagnosis, not for post-resection surveillance. As such, applying them to the latter without dedicated retraining and prospective evaluation would be exactly the type of unexamined dataset shift this review warns against. Recurrence and surveillance therefore sit low on the readiness ladder despite addressing one of the field’s most pressing unmet needs.

## 12. Emerging Technologies

Intellectual honesty requires separating what has been demonstrated in PDAC from what is extrapolated from broader oncology. **Foundation models**—those pretrained by self-supervision and adapted with limited labels—are beginning to influence pancreatic imaging and pathology, where transformer architecture and self-supervised pretraining improve data efficiency and cross-centre generalization, but PDAC-specific evidence is nascent [63]. **Generative AI and large language models** are being explored for documentation, structured reporting, terminology harmonization and tumour-board support, with early pancreatic evaluations showing feasibility alongside prompt sensitivity and divergence from experts on the more difficult decisions [36,63]. **Digital twins** built on foundation models are the most concrete emerging applications with real PDAC data, having shown subgroup-specific superiority over conventional stratification for chemotherapy-response predictions [33]. **Federated learning** and **synthetic data** are conceptually well matched to PDAC’s fragmented, privacy-constrained datasets and to the dataset-shift problem; however, published pancreatic-specific deployment remains sparse. **Agentic decision-support systems** are, for PDAC, aspirational. The unifying caution is that these technologies inherit—and sometimes amplify—the field’s core problems: a foundation model or digital twin still requires external and prospective validation, calibration, and workflow integration before it can be trusted to influence a decision.

It is worth being concise about why *federated learning* and *synthetic data* are singled out for PDAC. The field’s central methodological failure is dataset scarcity and shift—models trained at one centre collapse at another—and PDAC compounds this because its datasets are small, fragmented across institutions, and privacy-constrained. Federated learning, which trains a shared model across institutions without moving patient data, is therefore not a generic enthusiasm here but a direct structural response to the specific barrier that limits pancreatic AI. The caveat is that federated training does not by itself solve scanner, stain, or population heterogeneity, and pancreatic-specific federated deployments remain few. Synthetic data and generative augmentation are similarly matched to the small-sample problem but carry their own risk—models can learn artefacts of the generator rather than biology—so they demand the same external validation as any other method. *Agentic* and autonomous decision-support systems, which would chain retrieval, reasoning, and tool use to draft a management recommendation, are the furthest from evidence: the one concrete PDAC datapoint, the tumour-board LLM evaluation [36], shows exactly why, since a system with high aggregate accuracy silently failed on the neoadjuvant subgroup where the decision was hardest and most consequential. The recurring lesson is that emerging methods change what is *buildable* faster than they change what is *trustworthy*, and the readiness bar—external validation, calibration, prospective testing, and workflow fit—does not move because the architecture is new.

## 13. From Performance to Clinical Utility: The Translational Gap

**Figure 3:**
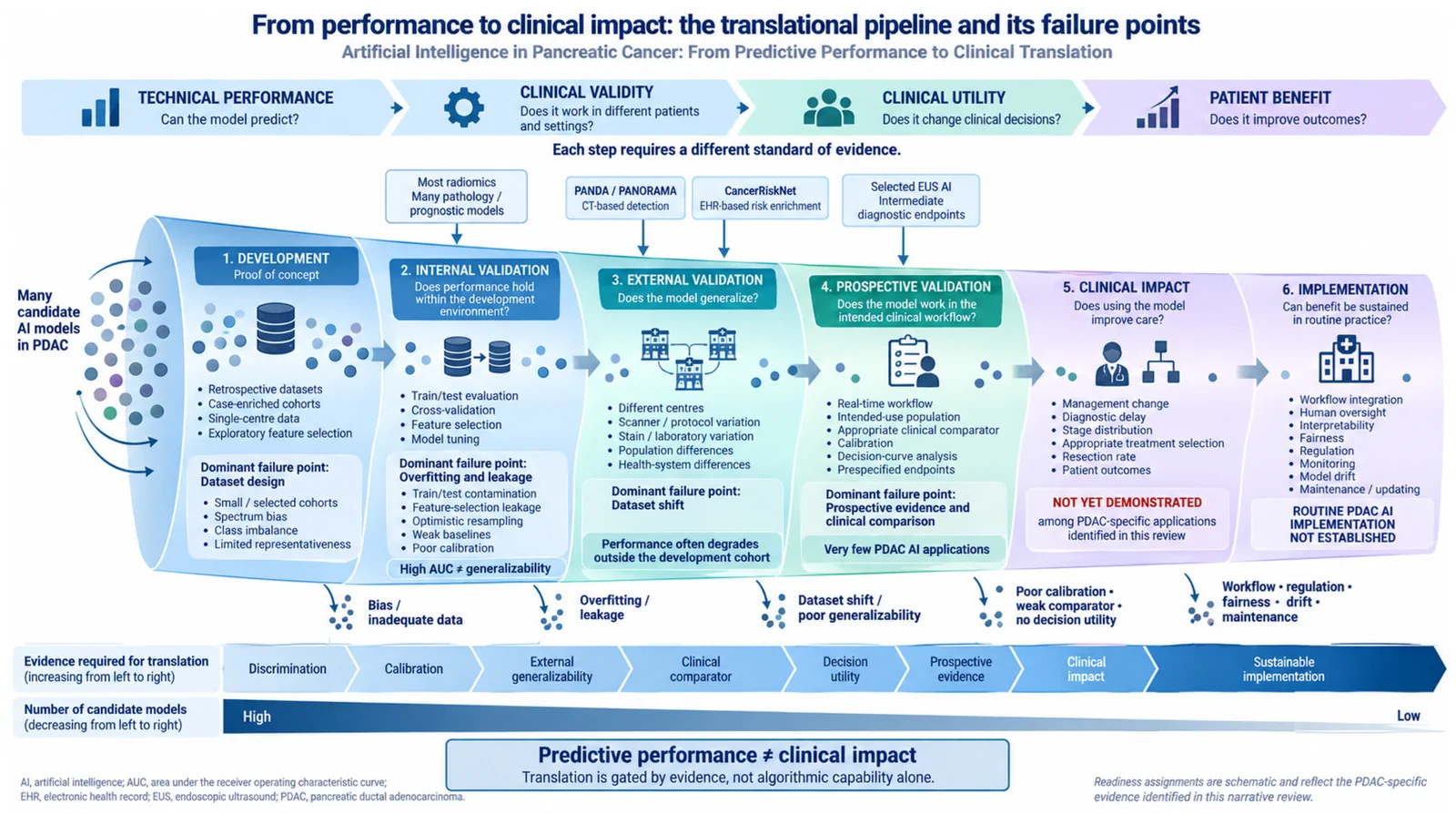
From performance to clinical impact: the translational pipeline and its failure points. Most pancreatic cancer AI is lost between high internal performance and demonstrated patient benefit. The pipeline shows the gates a model must pass and the dominant failure mode at each; width narrows to convey attrition. Very few PDAC applications have reached prospective validation, and among those identified in this review, none has demonstrated clinical impact—barriers to translation rather than algorithmic capability are now the concern.

Appraising the field domain by domain reveals one consistent pattern: reported technical performance and aggregate meta-analytic accuracy are high, yet almost nothing has moved from validation to demonstrated clinical utility (Figure 3). On the readiness ladder (Table 3), only two areas approach prospective or real-world scale: CT-based detection (PANDA’s real-world evaluation and PANORAMA’s international benchmark) and EHR-based risk enrichment (population-scale models and the END-PAC–anchored Early Detection Initiative) [22,23,24,25,45]—and even these lack an impact endpoint. A middle tier (computational pathology immune phenotyping, multimodal survival prediction, and the trial-validated treatment-selection biomarkers) has external or trial-embedded validation but no prospective clinical evaluation [28,29,30,31,32]. The largest tier, including most radiomics, most single-centre imaging and pathology classifiers, surgical planning and intraoperative AI, and recurrence/surveillance modelling—remains at the proof-of-concept or internal validation stage.

The reasons are structural, not algorithmic, and recur across sections. **Case-enriched, single-centre, retrospective development** inflates apparent performance through spectrum bias and misrepresents the false-positive burden under true prevalence—which is why the EHR meta-analysis’s sub-1% PPV [20] and REDMOD’s deliberate ~1:6 prevalence design [27] matter more than any major AUC. **Dataset shift**, including across scanners, vendors, stains, populations, and health systems, degrades external performance, as CancerRiskNet’s 0.88→0.71 cross-country drop demonstrates [24]. **Data leakage and overfitting** inflate internal metrics, especially in high-dimensional radiomics and omics with modest samples [11]. **Inadequate comparators** are pervasive: models are compared to no baseline, a weak baseline, or radiologists reading under artificial conditions, rather than to true clinical standards, such as multidisciplinary decisions, TNM, an established risk score, or the best simple model. **Calibration and decision-curve analysis** are usually absent; therefore, a model’s fitness for a real decision threshold is unknown. **Class imbalance** (low PDAC prevalence) makes accuracy and threshold-free AUC misleading, and **segmentation dependence** couples radiomics and many pathology models to an upstream step whose variability propagates downstream. Furthermore, **prospective evaluation and workflow integration are almost universally missing**: the number of PDAC-AI tools tested prospectively as decision aids, with an endpoint of changed management or improved outcome, is very small.

*Explainability, fairness, and regulation* are part of the same translational problem rather than separate topics. Several of the more rigorously validated systems (PANDA or immune-phenotype classifiers) are deliberately interpretable, outputting segmentations or spatial maps, which lowers barriers to trust in a disease where a false positive triggers invasive, costly, and anxiety-laden work-ups; opaque models face a higher bar. Training data are heavily skewed toward particular regions and health systems. Several landmark studies could not capture race or ethnicity, and generalizability to under-represented populations is largely untested [23,24]. PDAC datasets are fragmented and privacy-constrained (motivating federated approaches), and any deployed tool must meet device-regulatory requirements and be maintained against performance drift (continual-learning deployment, such as PANDA’s, begins to address drift, but most published models ignore this issue entirely [22]). These are not peripheral considerations; they are the actual rate-limiting steps between a high-performing model and a clinically used one. Representativeness should be examined explicitly along ethnicity, geography, age, sex, disease stage, healthcare setting, and socioeconomic status because demographic and health-system shifts can degrade both discrimination and calibration in ways that aggregate metrics hide.

**Data leakage.** Several failure modes deserve explicit mention because they recur in PDAC AI and are often invisible in headline metrics. Data leakage inflates internal performance whenever information from the test set influences training: splitting at the image or slice level rather than the patient level allows for repeated examinations of the same patient to fall into both sets; temporal leakage occurs when information from after the index time is available to the model; feature-selection and preprocessing leakage arise when selection, normalization, or harmonization is fitted on the whole dataset before splitting; and, most consequentially for early detection, incorporating post-diagnostic information into nominally prediagnostic models produces optimistic lead-time claims. Patient-level splitting, pipelines fitted inside cross-validation folds, and explicit temporal separation are minimum safeguards.

**Radiomics reproducibility.** This is a related, measurable problem. Handcrafted feature values are sensitive to scanner and vendor, slice thickness, reconstruction kernel, voxel resampling, intensity discretization, and segmentation variability; therefore, a “signature” can encode acquisition settings rather than biology. Minimum requirements therefore include image harmonization, demonstrated feature stability (test–retest or multi-reader segmentation), standardized preprocessing, phantom or technical validation where feasible, and adherence to radiomics standardization and reporting standards such as the Image Biomarker Standardization Initiative [58]. Few PDAC radiomics studies report these, which is one reason why meta-analytic radiomics quality scores remain low [19].

**Risks and unintended consequences.** Clinical AI also carries risks that accuracy metrics do not capture. False positives in a low-prevalence setting trigger invasive, costly, and anxiety-laden work-ups, whereas false negatives give false reassurance; both are amplified by automation bias, in which clinicians defer to a confident-seeming output, and by alert fatigue when low-value flags are frequent. Safe deployment therefore requires calibrated uncertainty communication, mechanisms for failure detection and human oversight, and prospective post-deployment monitoring for performance drift rather than one-off accuracy reporting—none of which the PDAC tools reviewed here have yet reported.

**Economic and implementation considerations.** Finally, clinical value depends on whether implementation justifies its cost. Beyond discrimination, adoption requires integration with picture-archiving (PACS) and electronic health record systems, computational and storage infrastructure, data annotation and curation efforts, software licencing and regulatory maintenance, and attention to the downstream costs of false-positive investigations. Formal economic evaluation, such as cost-effectiveness and budget-impact analyses, is essentially absent from the PDAC AI literature and should accompany a prospective clinical evaluation.

## 14. Future Directions and Research Agenda for Clinically Useful Pancreatic Cancer AI

The evidence points to a small number of high-yield priorities. First, **external and prospective validation should become the default**: the field needs fewer new single-centre models and more multicentre, prospective evaluations of the promising tools that already exist, including CT detection, EHR risk models, immune-phenotype pathology, and trial-validated treatment-selection biomarkers, with changed management and patient outcome endpoints rather than the AUC. Second, **early detection should be pursued as an integrated, cohort-based enterprise** combining EHR/new-onset-diabetes/germline enrichment, opportunistic imaging analysis, and circulating markers within defined high-risk populations, with performance always reported at intended-use prevalence [18,20,21,24,25,26,27,44,45,46,47,48,49,50,51]. Third, **multimodal work should be judged by incremental value and parsimony**, such as the smallest deployable analyte set validated against the best simple comparator [29]. Fourth, the field should **standardize reporting and benchmarking**, such as open FAIR benchmarks, prespecified analysis plans, calibration and decision-curve reporting, and adherence to STARD-AI/transparent-challenge standards, so that studies can become comparable and reproducible [10,23,37,38]. Fifth, **the prognostic/predictive distinction must be enforced**, with treatment-selection tools evaluated through treatment–interaction designs and, ultimately, prospective biomarker-stratified trials rather than survival associations [31,32]. Sixth, **workflow integration, calibration maintenance, and fairness auditing** should be treated as first-class research objectives because they—not model architecture—now determine whether a tool reaches patients.

## 15. Strengths and Limitations of This Review

This review has limitations that follow from its design. The search was deliberately non-systematic and narrative, and the study selection reflects expert judgement; therefore, relevant work may have been missed. Restriction to English-language, peer-reviewed sources indexed in the databases may have further excluded relevant work in other languages or nations, and the emphasis on landmark and high-performing studies risks over-representing positive results relative to negative or unpublished ones (i.e., publication and selective-reporting bias). Endpoints across the included studies were heterogeneous, such as discrimination, calibration, intermediate endpoints, and survival, precluding any formal pooling of our own; where pooled estimates appear, they are drawn from published meta-analyses. The field is evolving rapidly; therefore, some of the recent studies and preprints cited here await independent confirmation. Finally, the evidence-readiness framework and the maturity designations in Table 3 are an author-defined synthesis rather than a validated instrument, and their assignment is necessarily partly subjective; we have therefore made the criteria explicit (Figure 1, Section 3), applied them consistently, and framed conclusions as applying to the literature identified here. The review’s principal strengths are its explicit appraisal framework, its consistent separation of technical performance from clinical utility, and verification of cited performance figures against primary sources.

## 16. Conclusions

AI is being applied across the entire pancreatic cancer continuum, and in two domains: detection on CT and risk enrichment from routine clinical and imaging data, producing evidence that is strong enough to justify prospective clinical evaluation and, plausibly, near-term change in how and when PDAC is found. Elsewhere, such as in molecular discovery, computational pathology, multimodal integration, treatment-response prediction, surgery, and surveillance, the work is scientifically substantive and often technically impressive but, with a few externally validated exceptions, remains at the proof-of-concept or internal validation stage. Contemporary meta-analyses make the central tension quantitative: pooled accuracy is high, yet heterogeneity, low methodological quality, scarce external validation, and sub-1% positive predictive values at population prevalence show that aggregate performance overstates translational maturity. The field’s trajectory toward multimodal integration is among its more credible medium-term sources of clinically meaningful gains, provided it is disciplined by the incremental-value standard. Treatment prediction will advance only insofar as it respects the boundary between prognostic association and demonstrated treatment interaction. The decisive constraint is no longer algorithmic capability but translational evidence, such as external and prospective validation, calibration, appropriate comparators, and workflow integration. The question that should govern the next phase is, therefore, not whether a model can predict an endpoint, but whether it produces information that is reliable, generalizable, and actionable enough to improve a decision that changes a patient’s outcome. This prediction should be graded on that standard; only a minority of applications can yet answer it, and identifying which ones—and what each still needs—is the purpose of this review.

## Figures and Tables

**Table 1:** Artificial intelligence across the pancreatic cancer continuum.

Domain/Stage	Clinical or Research Question	Principal Data Modality	Representative AI Approach	Potential Application	Readiness *
Biological discovery	What drives PDAC biology and immunosuppression?	Genomics, transcriptomics, single-cell, spatial, proteomics	Clustering; ML survival modelling; virtual screening	Target/biomarker discovery; drug repurposing	Development/internal
Risk enrichment	Who is at high enough risk to warrant testing?	EHR diagnostic-code sequences; new-onset-diabetes metrics	Transformer/sequence models; transparent risk scores	Surveillance-cohort enrichment	Externally validated; prospective ongoing
Early detection (imaging)	Is there subclinical disease on routine imaging?	Non-contrast and contrast CT; pancreas segmentation	Radiomics; ML; DL (nnU-Net, transformers)	Opportunistic/prediagnostic detection	Externally validated → early real-world
Diagnosis and differential	PDAC vs. other lesions?	CT, MRI, EUS, cyst fluid	CNNs; multimodal models	Triage; fewer missed/mischaracterized lesions	Internally validated; some prospective (EUS)
Staging and resectability	Resectable, borderline, or locally advanced?	Contrast CT; segmentation	DL segmentation; radiomic nomograms	Vessel-contact quantification; planning	Internal validated
Computational pathology	Biology/outcome from tissue morphology	Whole-slide H&E	CNN/transformer WSI analysis	Immune phenotyping; response readouts	Internally validated; independent biological correlation
Multi-omics/multimodal	Does integration beat single sources?	Clinical + imaging + pathology + omics + plasma	Ensemble ML; parsimonious selection; digital twins	Prognosis; preoperative decision support	Externally validated (survival)
Treatment selection	Which regimen benefits this patient?	Bulk transcriptomics; histology; clinico-genomics	Drug-specific signatures; computational histology	FOLFIRINOX vs. gemcitabine/nab-paclitaxel	Trial-embedded retrospective validation
Surgery and perioperative	Resectability; will a complication occur?	Preoperative CT + clinical; intraoperative video	Ensemble ML/DL; computer vision; LLMs	Planning; POPF/futility prediction; guidance	Internally validated; intra-op = development
Prognosis and surveillance	Outcome; is disease recurring?	Pathology, imaging, molecular, longitudinal	ML/DL survival models; radiomics	Stratification; earlier recurrence detection	Mixed; surveillance largely absent
Emerging (FM/LLM/twins)	Generalizable, integrative, generative support	Heterogeneous multimodal	Foundation models; LLMs; digital twins; federated learning	Data-efficient models; reporting; response prediction	Development (PDAC-specific)

* Readiness ladder: development → internal validation → external validation → prospective validation → clinical impact → implementation. EHR, electronic health record; EUS, endoscopic ultrasound; WSI, whole-slide image; POPF, postoperative pancreatic fistula; FM, foundation model; LLM, large language model.

**Table 2:** Landmark and recent high-impact studies and syntheses in pancreatic cancer AI.

Study	Year	Task	Study Design/Cohort	Validation	Comparator	Key Result	Main Limitation	Study Design and Readiness Rationale
**Meta-analyses (aggregate evidence)**								
Alidina et al., *J. Gastrointest. Surg.* [18]	2026	Radiomics early detection	15 studies, 14,688 patients	Meta-analytic	—	Pooled sensitivity 0.88, specificity 0.93	I^2^ ≈ 88–95%; calls for prospective evaluation	Systematic review/meta-analysis; aggregate accuracy only, high heterogeneity, no external/prospective data—not a readiness statement for any single tool.
Zhang et al., *J. Med. Internet. Res.* [19]	2025	Radiomics PDAC vs. mass-forming pancreatitis	24 studies, 14,406 cases	Meta-analytic	—	Pooled sensitivity 0.92, specificity 0.90	Mean radiomics quality ~9/36; poor external validation/reproducibility	Systematic review/meta-analysis; high pooled accuracy but low radiomics quality and scarce external validation—internal-to-external at best.
Makiev et al., *Cancers* [20]	2026	EHR-based AI risk	19 studies	Meta-analytic	—	Pooled AUC 0.785 (NN 0.826)	PPV often <1%; extreme heterogeneity	Systematic review/meta-analysis; population-scale discrimination but sub-1% PPV—supports risk enrichment, not population screening.
Filis et al., *Eur. J. Cancer* [21]	2026	Liquid biopsy + AI early detection	109 studies (pancreatic *n* = 16)	Meta-analytic	—	Specificity 94–99%	Stage I sensitivity 44–91%	Systematic review/meta-analysis; high specificity, variable stage-I sensitivity—early detection unproven at stage I.
**Landmark primary studies**								
Cao et al. (PANDA), *Nat. Med.* [22]	2023	Detection and subtyping	Non-contrast CT; 3208 train; 6239 multicentre; 20,530 real-world	Multicentre external + real-world (retrospective)	Radiologist readers; contrast-CT reports	AUC 0.986–0.996; +34.1% sens vs. mean reader; 99.9% real-world specificity	East-Asian-predominant; not prospective; no impact endpoint	Retrospective multicentre + consecutive real-world, reader-benchmarked; geographic external validation; no prospective/impact endpoint → external/real-world.
Alves et al. (PANORAMA), *Lancet Oncol.* [23]	2026	PDAC detection	Contrast CT; open benchmark; 391-case reader subset	International multicentre; paired reader study	68 radiologists, 12 countries	AUC 0.92 vs. 0.88 (non-inferior and superior); fewer false positives	Controlled reading; no stage/resection/survival endpoint	International multicentre paired reader study (non-inferiority then superiority); controlled reading, no outcome endpoint → external, reader-benchmarked.
Placido et al. (CancerRiskNet), *Nat. Med* [24]	2023	Risk prediction (36-mo)	EHR code sequences; ~6M (DK) + ~3M (US-VA)	Cross-country external	Age/clinical baselines	AUC 0.88 (0.83 excl. last 3 mo)	Dropped to 0.71 cross-system (dataset shift)	Retrospective EHR; cross-country external validation with performance drop requiring retraining → external; prospective pending.
Sharma et al. (END-PAC), *Gastroenterology* [25]	2018	Risk enrichment (new-onset diabetes)	1561 discovery; 1096 validation	Independent internal	Clinical judgement	AUC 0.87; score ≥ 3 → ~4.4× prevalence	Small case numbers; anchors prospective EDI	Retrospective derivation + independent internal validation; anchors a prospective trial (EDI) → internal → prospective in progress.
Korfiatis et al., *Gastroenterology* [26]	2023	Diagnostic + prediagnostic detection	Automated 3D-CNN; 3014 CTs + external	External public + bootstrapped NOD prevalence	—	Diagnostic AUROC 0.97; prediagnostic 0.91 at 475 d lead	Retrospective case–control; not prospective	Retrospective case–control; external public dataset + simulated-prevalence testing → external, not prospective.
Mukherjee et al. (REDMOD), *Gut* [27]	2026	Prediagnostic detection	Radiomics; low-prevalence (~1:6) design	External specificity; longitudinal	Radiologists	AUC 0.82; ~2× radiologist sensitivity; 475 d lead	Case–control; not prospective	Retrospective case–control; external specificity + longitudinal stability at ~1:6 prevalence → external, not prospective.
Kim et al., *JAMA Surg.* [28]	2025	Immune phenotyping/prognosis	H&E WSI; 304 resected PDAC	Single-centre; TCGA correlation	TNM stage	Immune phenotype independent OS/RFS predictor	Single-centre; dated adjuvant therapy; no biopsy validation	Single-centre retrospective; independent biological (TCGA) correlation, no external clinical cohort → internal validation.
Osipov et al. (Molecular Twin), *Nat. Cancer* [29]	2024	Survival prediction	Multi-omic; 74 pilot + 4 external	4 independent external cohorts	CA 19-9	589-feature model ≈ full 6363; plasma protein > CA 19-9	Small pilot; single time-point	Retrospective; external validation across four cohorts; small pilot → external (prognostic).
Schuurmans et al., *Eur. Radiol.* [30]	2025	Multimodal prognostication	Clinical + CT; 401 dev; 361 external	External (Dutch + Spanish)	TNM; unimodal	Beat TNM; AUC 0.637 internal/0.571–0.675 external	Modest discrimination; retrospective	Retrospective; geographic external validation (Dutch + Spanish); modest discrimination → external (modest gain).
Fraunhoffer et al. (Pancreas-View), *Ann. Oncol.* [31]	2024	Predictive treatment selection	Transcriptomic + TME	Randomized-trial tissue (PRODIGE-24, *n* = 343)	Performance-status choice	Predicted-sensitive mFFX DFS 50.0 mo (HR 0.31)	Trial-derived retrospective; not prospective decision aid	Retrospective analysis of randomized-trial tissue; not prospective biomarker-guided → trial-embedded retrospective.
Beaufils et al. (PANCprAId), *J. Clin. Oncol.* [32]	2026	Predictive biomarker (interaction)	Histology DL; 231 train	External (PRODIGE-24/PA6, *n* = 313)	GEM vs. mFFX arms	**Treatment interaction *p* = 0.003 (DFS), 0.001 (CSS)**	Retrospective validation; awaits prospective trial	Retrospective; formal treatment-interaction test on randomized-trial tissue → trial-embedded retrospective (predictive-design exemplar).
Griffiths et al. (FarrSight), *Front. Artif. Intell.* [33]	2026	Digital-twin response prediction	Multi-omic; COMPASS (*n* = 206)	Trial cohort	Moffitt subtype	Basal-like mFFX response AUC 72.3% vs. 44.8%	Small; benefit confined to one subtype/regimen	Trial-cohort validation; subgroup/regimen-confined, small → internal-to-external (frontier).
Lee et al., *Sci. Rep.* [34]	2024	POPF prediction	Preoperative CT + clinical; *n* = 1333	Temporal internal	Prior scores	AUC 0.75 (all POPF); 0.68 (CR-POPF)	Single-centre; modest AUC	Single-centre; temporal internal validation → internal.
Ahmed et al., *J. Surg. Res.* [35]	2025	Surgical futility (early death)	NCDB registry; *n* = 66,331	Held-out internal	Logistic regression	GB AUC 0.689	Registry granularity; moderate AUC	Registry (NCDB); held-out internal validation → internal.
Mergen et al., *BMC Med. Inform. Decis. Mak.* [36]	2026	LLM tumour-board support	42 PDAC cases	Against board decisions	MDT ground truth	Prompt design decisive; diverged on resectability/metastasis	Small; forced-choice; not deployed	Reader-type evaluation against board decisions in 42 cases; not deployed → development/proof-of-concept.

OS, overall survival; RFS, recurrence-free survival; DFS, disease-free survival; CSS, cancer-specific survival; CR-POPF, clinically relevant POPF; TME, tumour microenvironment; MDT, multidisciplinary team; NN, neural network; GEM, gemcitabine; mFFX, modified FOLFIRINOX; NCDB, National Cancer Database; NOD, new-onset diabetes.

**Table 3:** Evidence-readiness matrix for AI applications in pancreatic cancer. Each application is scored on four orthogonal dimensions before an overall maturity level is assigned because the dimensions are not interchangeable.

Application	Validation (Best Achieved)	Comparator (Adequacy)	Actionability (Would It Change a Decision?)	Impact (Shown to Improve care?)	Overall Maturity	Highest Evidence Tier Reached *
EHR-based risk enrichment	External (cross-country; performance drop)	Clinical/actuarial baselines—adequate	High—defines surveillance cohorts	Not demonstrated; prospective trial ongoing	External validation; prospective trial ongoing	Clinical validity (external); utility prospective-pending
CT-based detection (opportunistic)	Multicentre external + retrospective real-world	Radiologist pools—strong	High—earlier detection/triage	Not demonstrated (no stage/resection/survival endpoint)	External validation + retrospective real-world evaluation	Clinical validity (external/real-world); clinical utility not yet established
Prediagnostic radiomics	External specificity; low-prevalence design	Radiologists—adequate	Moderate–high (surveillance)	Not demonstrated	External validation (specificity); not prospective	Technical performance → early clinical validity
EUS AI	Single-centre RCTs (intermediate endpoints)	Standard EUS—adequate	Moderate	Not demonstrated (no outcome endpoint)	Prospective validation of intermediate endpoints	Clinical validity (prospective; intermediate endpoints)
Computational pathology (immune phenotype)	Single-centre; independent biological correlation (TCGA); no external clinical cohort	TNM stage—adequate	Moderate (complements staging)	Not demonstrated	Internal validation/independent biological correlation	Clinical validity (internal); clinical utility not yet established
Molecular subtyping	External, multi-cohort	Existing subtype schemes—often absent	Low–moderate (rarely changes management)	Not demonstrated	External validation; low actionability	Clinical validity (external); low actionability
Multimodal survival prediction	External (modest AUC)	TNM, unimodal—adequate	Moderate	Not demonstrated	External validation (modest incremental gain)	Clinical validity (external, modest)
Treatment-response prediction	Randomized-trial tissue; treatment interaction (PANCprAId)	Regimen arms/performance status—strong	High if confirmed	Not demonstrated (no prospective biomarker-guided trial)	External/trial-embedded retrospective validation	Clinical validity (trial-embedded); predictive design
Resectability/surgical planning	Internal	Radiologists—partial	High	Not demonstrated	Internal validation	Technical performance/internal validity
Postoperative risk (POPF, futility)	Internal/registry	Often weak (not vs. Fistula Risk Score)	Moderate	Not demonstrated	Internal validation/registry	Technical performance/internal validity
Recurrence/surveillance	Single-centre, rare external	Standard model—rarely tested	Moderate–high (unmet)	Not demonstrated	Development–internal validation	Technical performance (development)
Foundation models/LLMs/digital twins	Nascent, PDAC-specific	Conventional stratification—occasional	Uncertain	Not demonstrated	Development (PDAC-specific)	Technical performance (development)

* “Overall maturity” is expressed using the evidence-readiness ladder defined in Section 3 (development → internal validation → external validation → prospective validation → clinical impact → implementation). In the Impact column, “Not demonstrated” indicates that no evidence of demonstrated clinical impact was identified in this review (see Methods), not formal proof of its absence. Evidence tiers (ascending): technical performance (discrimination/accuracy); clinical validity (associates with the intended target in the intended population, ideally externally); clinical utility (changes a decision beneficially); workflow impact (deployable and used in practice); and patient-level outcome (improves stage, resection, survival, or quality of life). Abbreviations: AI, artificial intelligence; AUC, area under the curve; CT, computed tomography; EHR, electronic health record; EUS, endoscopic ultrasonography; LLM, large language model; PDAC, pancreatic ductal adenocarcinoma; POPF, postoperative pancreatic fistula; RCT, randomized controlled trial; TCGA, The Cancer Genome Atlas; TNM, tumour–node–metastasis.

**Table 4:** Illustrative effect of pre-test prevalence on positive and negative predictive value for fixed sensitivity and specificity.

Pre-Test Prevalence	PPV (Se 90%, Sp 95%)	NPV (Se 90%, Sp 95%)	PPV (Se 90%, Sp 99%)	NPV (Se 90%, Sp 99%)
0.1% (population)	1.8%	>99.9%	8.3%	>99.9%
1%	15.4%	99.9%	47.6%	99.9%
5%	48.6%	99.4%	82.6%	99.5%
10% (high-risk/enriched)	66.7%	98.8%	90.9%	98.9%

Values are illustrative, computed from Bayes’ theorem at two hypothetical operating points rather than drawn from a specific study. At population-screening prevalence (<1%), even 99% specificity yields a positive predictive value below 50%, whereas negative predictive value stays high throughout—quantifying why case–control accuracy overstates screening performance and why electronic health record models report sub-1% PPV at population prevalence [18]. Se, sensitivity; Sp, specificity; PPV, positive predictive value; NPV, negative predictive value.

## Data Availability

No new data were created or analyzed in this study. Data sharing is not applicable to this article.

## Supplementary Materials

The following supporting information can be downloaded at: https://www.mdpi.com/article/10.3390/cancers18203253/s1, Figure S1: Conceptual (non-numeric) study-selection process; Table S1: Database-specific search strategies (reconstructed and run for transparency during the revision; most recent search October 2026).

## Abbreviations

The following abbreviations are used in this manuscript:

AI	Artificial Intelligence
AUC	Area under the receiver operating characteristic curve
CA 19-9	Carbohydrate antigen 19-9
CNN	Convolutional neural network
CT	Computed tomography
DL	Deep learning
EHR	Electronic health record
EUS	Endoscopic ultrasonography
FAIR	Findable, Accessible, Interoperable, and Reusable
FM	Foundation model
FOLFIRINOX	5-fluorouracil, leucovorin, irinotecan, and oxaliplatin
GEM	Gemcitabine
H&E	Hematoxylin and eosin
LLM	Large language model
mFFX	Modified FOLFIRINOX
ML	Machine learning
MRI	Magnetic resonance imaging
NOD	New-onset diabetes
OS	Overall survival
PANDA	Pancreatic cancer detection with artificial intelligence
PDAC	Pancreatic ductal adenocarcinoma
POPF	Postoperative pancreatic fistula
PPV	Positive predictive value
STARD-AI	Standards for Reporting Diagnostic Accuracy Studies–Artificial Intelligence
TNM	Tumor, node, metastasis
WSI	Whole-slide imaging
AUROC	Area under the receiver operating characteristic curve
CAD	Computer-aided detection
IBSI	Image Biomarker Standardization Initiative
NPV	Negative predictive value
PACS	Picture archiving and communication system
RCT	Randomized controlled trial
SPN	Solid pseudopapillary neoplasm
TCGA	The Cancer Genome Atlas
