# Supplementary material for: Artificial Intelligence in Pancreatic Cancer: From Predictive Performance to Clinical Translation
Source: Cancers (Basel). 2026 Oct 9;18(20):3253. doi: 10.3390/cancers18203253 (PMC13702653; doi:10.3390/cancers18203253)
Supplement: Supplementary file 1 [file cancers-18-03253-s001.zip › cancers-4611456-supplementary.pdf]

Supplementary Material

Artificial Intelligence in Pancreatic Cancer: From Predictive Performance to Clinical Translation

Manuscript ID: cancers-4611456

Note. This review is a narrative, non-systematic synthesis. The database search strategies below are provided to make the basis of study selection transparent. They were reconstructed and run for transparency during the revision—the most recent search was performed in October 2026—and are not presented as a prospectively preserved search string or a systematic-review protocol. Searches emphasised English-language, peer-reviewed work published between January 2022 and 2026, supplemented by seminal earlier studies and by citation chaining from recent meta-analyses, consensus statements and landmark primary studies. Study identification and selection were carried out by members of the author team as part of an expert-curated process rather than by independent dual screening; where several reports described the same cohort, the most complete or most recent was used. The number of records screened was not prospectively recorded, and no numeric screening counts are reported, consistent with the non-systematic design (Supplementary Figure S1).

Supplementary Table S1. Database-specific search strategies (reconstructed and run for transparency during the revision; most recent search October 2026).

Database / platform	Search strategy	Limits
PubMed / MEDLINE	("Pancreatic Neoplasms"[MeSH] OR "pancreatic cancer"[tiab] OR "pancreatic ductal adenocarcinoma"[tiab] OR PDAC[tiab]) AND ("Artificial Intelligence"[MeSH] OR "artificial intelligence"[tiab] OR "machine learning"[tiab] OR "deep learning"[tiab] OR radiomics[tiab] OR radiogenomics[tiab] OR "computational pathology"[tiab] OR "digital pathology"[tiab] OR "multi-omics"[tiab] OR multimodal[tiab] OR "neural network*"[tiab] OR "foundation model*"[tiab] OR "large language model*"[tiab] OR "generative AI"[tiab])	English; 2022–2026 emphasis; humans. Seminal pre-2022 records added by citation chaining.
Web of Science Core Collection	TS=("pancreatic cancer" OR "pancreatic ductal adenocarcinoma" OR PDAC) AND TS=("artificial intelligence" OR "machine learning" OR "deep learning" OR radiomics OR radiogenomics OR "computational pathology" OR "digital pathology" OR "multi-omics" OR multimodal OR "neural network*" OR "foundation model*" OR "large language model*" OR "generative AI")	Timespan 2022–2026; language = English; document types = Article, Review.
Scopus	TITLE-ABS-KEY("pancreatic cancer" OR "pancreatic ductal adenocarcinoma" OR PDAC) AND TITLE-ABS-KEY("artificial intelligence" OR "machine learning" OR "deep learning" OR radiomics OR radiogenomics OR "computational pathology" OR "digital pathology" OR "multi-omics" OR multimodal OR "neural network*" OR "foundation model*" OR "large language model*" OR "generative AI") AND PUBYEAR > 2021	LIMIT-TO(LANGUAGE,"English"); LIMIT-TO(DOCTYPE,"ar","re").
Google Scholar	("pancreatic cancer" OR PDAC) AND ("artificial intelligence" OR "machine learning" OR "deep learning" OR radiomics OR "digital pathology" OR "foundation model" OR "large language model"), run per domain and combined with the domain-specific terms below; used chiefly for citation chaining and coverage checks.	English; used for citation chaining and coverage checks; 2022–2026 emphasis.
Domain-specific terms (combined with the disease and AI	Early detection / risk: "new-onset diabetes", "electronic health record", "liquid biopsy", "circulating tumor DNA", screening, "high-risk". Imaging: "computed tomography", MRI, PET, "endoscopic ultrasound" / EUS, segmentation, staging,	As above.

blocks above, by section)	resectability, "vascular involvement". Pathology: "whole slide imaging", grading, "tumor microenvironment", spatial. Molecular: genomic*, transcriptomic*, proteomic*, metabolomic*, "single-cell", "spatial transcriptomics", subtyp*. Treatment: FOLFIRINOX, gemcitabine, neoadjuvant, chemotherapy, radiotherapy, immunotherapy, "treatment response". Surgery / perioperative: "surgical planning", "pancreatic fistula", complication*, "delayed gastric emptying". Prognosis / surveillance: survival, recurrence, surveillance, prognosis. Emerging: "foundation model", "large language model", "vision-language", "federated learning", "digital twin", "self-supervised".	

PDAC, pancreatic ductal adenocarcinoma; MeSH, Medical Subject Headings; tiab, title/abstract; TS, topic; EUS, endoscopic ultrasonography. Asterisks denote truncation/wildcard operators. Strategies were adapted to each platform's syntax; the disease and AI concept blocks were held constant and combined with the domain-specific terms listed in the final row for each section of the review.

Supplementary Figure S1. Conceptual (non-numeric) study-selection process.

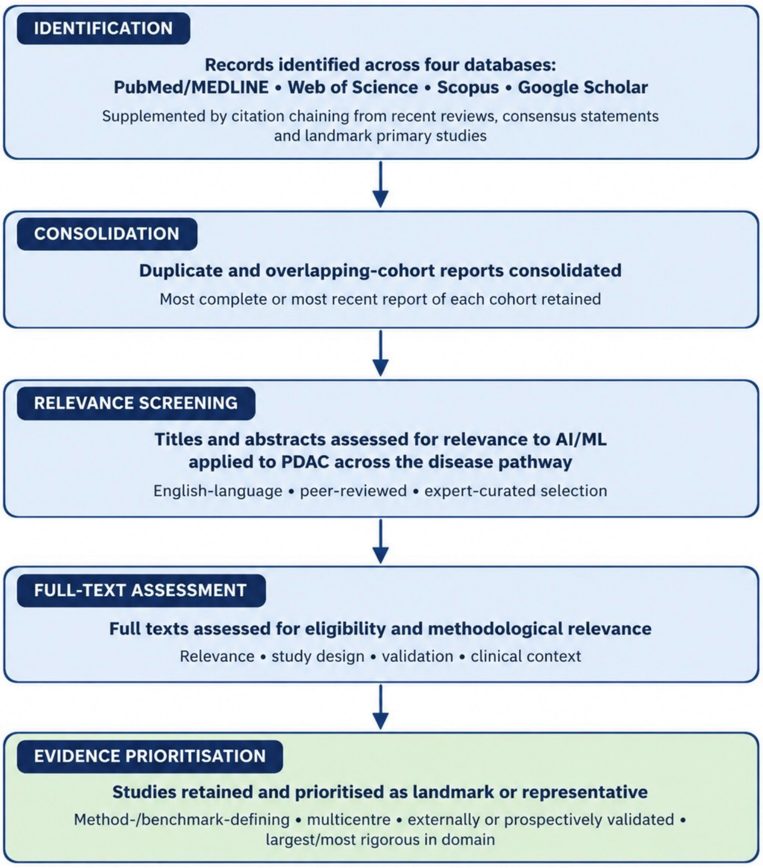

Because this is a narrative, non-systematic review, the schematic is deliberately conceptual and reports no record counts. It depicts the sequence of identification, consolidation of duplicate/overlapping cohorts, relevance screening, full-text assessment and prioritisation of landmark or representative studies described in Section 2 of the main text.
